# A simulation study of in-beam visualization system for proton therapy by monitoring scattered protons

**DOI:** 10.3389/fmed.2023.1038348

**Published:** 2023-07-14

**Authors:** Shogo Sato, Hiromu Yokokawa, Mana Hosobuchi, Jun Kataoka

**Affiliations:** Faculty of Science and Engineering, Waseda University, Tokyo, Japan

**Keywords:** proton therapy, dose range verification, deep learning, scattering protons, CT utilization, scintillation detectors, current readout

## Abstract

Recently, in-beam positron emission tomography (PET) has been actively researched for reducing biological washout effects and dose monitoring during irradiation. However, the positron distribution does not precisely reflect the dose distribution since positron production and ionization are completely different physical processes. Thus, a novel in-beam system was proposed to determine proton dose range by measuring scattered protons with dozens of scintillation detectors surrounding the body surface. While previous studies conducted a preliminary experiment with a simple phantom, we simulated more complex situations in this paper. Especially, we conducted three stepwise simulation studies to demonstrate the feasibility of the proposed method. First, a simple rectangular phantom was reproduced on simulation and irradiated with protons for obtaining current values and Monte Carlo (MC) dose. Next, we trained a deep learning model to estimate 2-dimensional-dose range (2D-DL dose) from measured current values for simulation (A). We simulated plastic scintillators as detectors to measure the scattered protons. Second, a rectangular phantom with an air layer was used, and 3D-DL dose was estimated in simulation (B). Finally, a cylindrical phantom that mimics the human body was used for confirming the estimation quality of the simulation (C). Consequently, the position of the Bragg peak was estimated with an error of 1.0 mm in simulation (A). In addition, the position of the air layer, as well as the verifying peak position with an error of 2.1 mm, was successfully estimated in simulation (B). Although the estimation error of the peak position was 12.6 mm in simulation (C), the quality was successfully further improved to 9.3 mm by incorporating the mass density distribution obtained from the computed tomography (CT). These simulation results demonstrated the potential of the as-proposed verification system. Additionally, the effectiveness of CT utilization for estimating the DL dose was also indicated.

## 1. Introduction

Proton therapy, which was proposed by Wilson (1), was first put to practical use in 1954 for cancer treatment at Lawrence Berkeley Laboratory (2). Proton therapy has received extensive attention in radiation oncology due to its high dose concentration, and the increase in the number of patients being treated. However, dose range uncertainties may cause significant damage to tumors close to critical organs because of its high dose concentration. Thus, a reliable in-beam monitoring system is essential to achieve both safety of the treatment and its effectiveness in eradicating tumor cells. Conventionally, the dose range was verified after the irradiation, by measuring the annihilation gamma rays from positrons with positron emission tomography (PET) (3–11). PET monitoring is one of the most well-studied methods for dose range verification and has already been exploited clinically (12). Recently, in-beam PET (13–17) has been actively researched for reducing biological washout effects and dose monitoring during irradiation. However, the correlation between the positron distribution and the proton dose range is not so straightforward since positron production and ionization are completely different physical processes. Therefore, some researchers have attempted to verify the dose range based on the maximum likelihood expectation maximization (MLEM) (18, 19) or machine learning (20–22).

As with other methods, the prompt gamma rays, which are produced by the de-excitation of the elements such as ^12^C^*^ and ^11^B^*^ upon being irradiated by protons, have been measured by a Compton camera (23–25) and slit camera (26). The prompt gamma rays are emitted immediately after irradiation, and their distribution correctly reflects the proton dose range. Moreover, other researchers have estimated the dose range by observing Cherenkov light derived from secondary electrons (27–31). Although these solutions utilizing gamma rays or Cherenkov light have the potential to verify the dose range precisely, devices and reconstruction processes tend to be expensive and complex.

To overcome such situations, there is a growing demand for an in-beam and simple verification system to determine dose range with fewer ambiguities. Thus, Sato et al. (32) proposed a novel in-beam verification system to observe scattered protons by placing dozens of scintillation detectors surrounding the body surface. To achieve system simplicity, they integrated the output pulses from each detector and read them as a current value. For further improvement, the dose range was estimated by deep learning algorithms. While previous studies conducted a preliminary experiment using a 70 MeV proton beam irradiated onto a simple polystyrene phantom, we simulated situations closer to the practical application by using a water phantom irradiated with 200 MeV protons. Moreover, to mitigate the risk of over-fitting by the machine-learning model due to overly simple phantom geometry, we performed simulations with progressively increasing levels of complexity.

Measurement systems with a similar concept were proposed for heavy particle therapy (33–37), but these systems are designed to measure secondary charged particles produced by nuclear reactions with heavy particles. In contrast, our system directly measures scattered protons, the same as the injected particles. Despite their resemblance, these systems have completely different purposes and principles.

In this paper, we conducted three stepwise simulations to demonstrate the feasibility of the proposed method. First, a simple rectangular phantom was reproduced on simulation and irradiated with protons for obtaining current values and Monte Carlo (MC) dose. Next, we trained a deep learning model to estimate 2-dimensional-dose range (2D-DL dose) from the output current of the detectors placed at different positions on the phantom surface. Second, a rectangular phantom with an air layer was used, and 3D-DL dose was estimated. Finally, a cylinder phantom with an internal structure was used, and we estimated the 3D-DL dose in the same manner as the rectangular phantom. The remainder of this article is organized as follows: In Section 2, the verification details of the simulation and estimating model are presented. Estimating results of DL dose are presented in Section 3. In Section 4, we describe the effectiveness of computed tomography (CT) utilization and system optimization. In Section 5, the conclusions are presented.

## 2. Method

**Figure 1:**
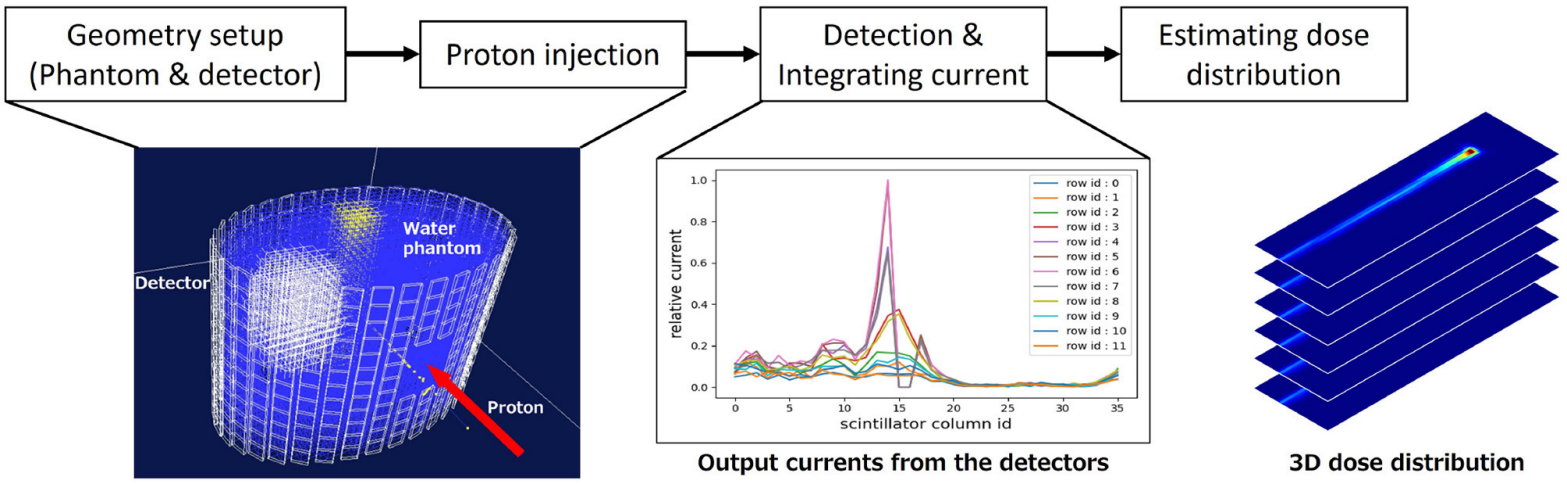
Flowchart of dose range verification system. First, we set the phantom and place the scintillation detectors surrounding the phantom surface. Second, we irradiate the proton beam and measure the output current from each detector. Finally, the proton dose range is estimated by deep learning.

In this section, we firstly summarize the proton dose range verification system. The simple flow chart of DL dose estimation is shown in Figure 1. Secondly, the simulation setup and deep learning models are described. In this paper, we simulated the geometries and proton beam on Geant4 (38, 39), which is a simulation toolkit developed by CERN, and has been used for the dose range verification of proton therapy (40–42). In this study, we employed "TestEM7", one of the extended example codes, as the nuclear physics process for Geant4 simulations. In particular, we utilized three distinct physics models, namely, PhysListEmStandard, PhysListEmStandardNR, and PhysListEmStandardSS.

### 2.1. Proton dose range verification system

Sato et al.(32) proposed a novel in-beam visualization system of 3D dose range for precision proton therapy. In this system, particles leaked outside the human body were monitored by dozens of scintillation detectors, e.g. 36 × 12 scintillation detectors coupled with plastic scintillator and multi-pixel photon counter (MPPC), surrounding the body surface. The energy deposited by the scattered particles, mainly scattered protons, was interpreted as current by integrating the pulses for system simplification. The cross-section of proton scattering and proton dose range follow the Rutherford formula in Eq. 1 and Bethe-Bloch formula, respectively.


(1) $$\begin{array}{l} \frac{d\sigma }{d\Omega }={\left(\frac{Ze^{2}}{4E}\right)}^{2}\frac{1}{\sin^{4}\theta }, \end{array}$$

where *Z*, *e*, and θ represent the atomic number of irradiated materials, the particle charge, and the scatter angle, respectively. Thus the number of scattered protons increases as ∝*E*^−2^, although the average energy of each scattered proton becomes small. Since the actual radiation dose deposited by scattered protons is a product of the number and energy per scattered proton, it has the potential to estimate the rough dose range in a relatively easy and cost-effective way. For further improvement, the precise dose range was estimated by incorporating deep learning. The previous study was restricted to verifying their results with simple rectangular polystyrene phantoms. In contrast, we employed a water phantom with progressively increasing levels of complexity.

### 2.2. Simulated verification summary

**Figure 2:**
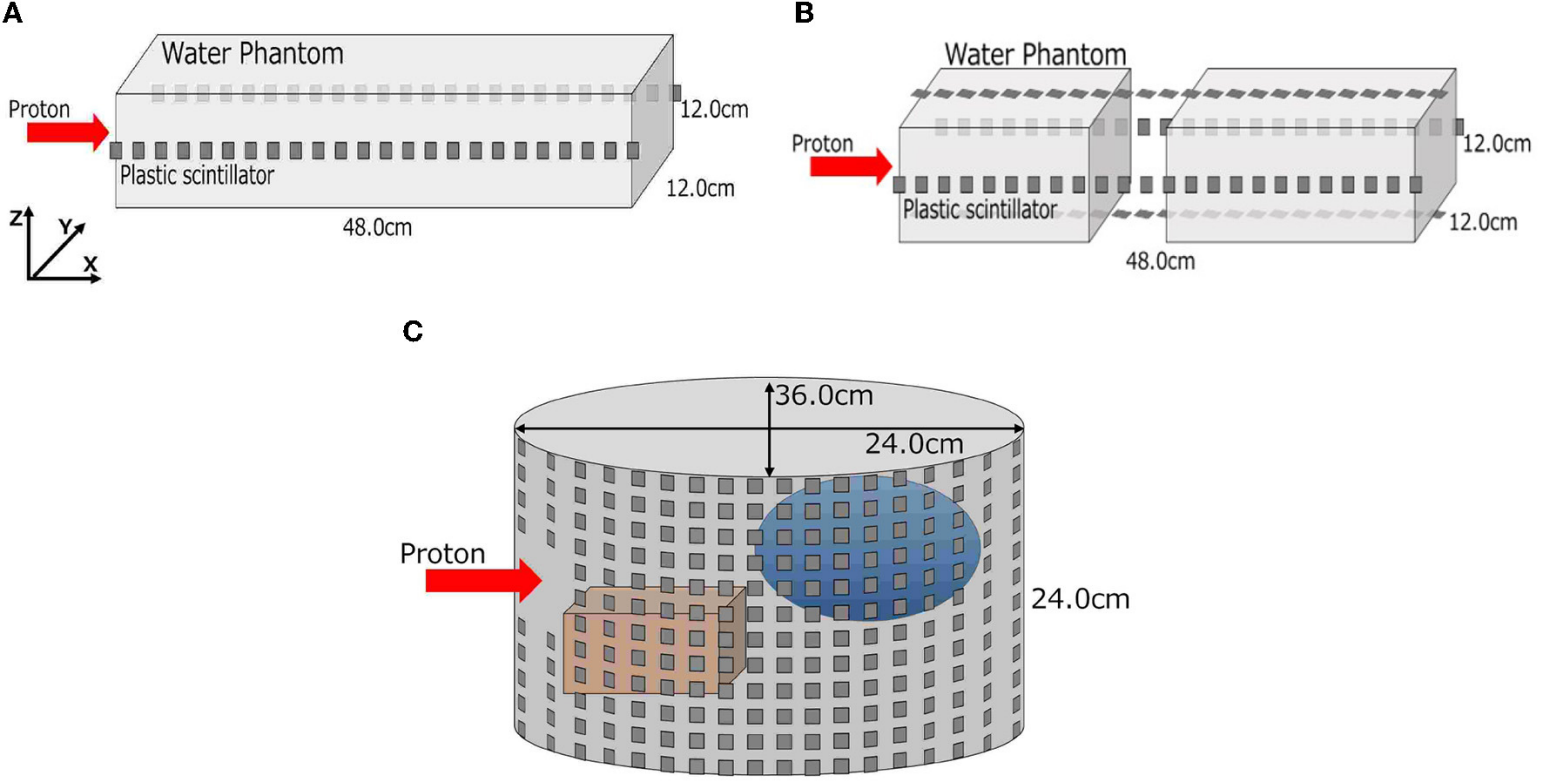
Geometries of three-stepwise simulation. A simple rectangular phantom in simulation (A), a rectangular phantom with an air layer in simulation (B), and a cylinder water phantom with internal structures in simulation (C) are depicted. **(A)** Simulation (A), **(B)** simulation (B), and **(C)** simulation (C).

We conducted three stepwise simulations to demonstrate the feasibility of the proposed method. These simulated conditions are summarized in Table 1, and the geometry in each simulation is shown in Figure 2. First, a simple rectangular phantom, which consists of water (1.0 g/cm^3^), was irradiated with protons in simulation (A) for obtaining current values and MC dose. We placed 24 scintillation detectors on both sides of the phantom surface. Next, we trained a deep learning model to estimate 2D-DL dose from the output current of the detectors. Second, a rectangular water phantom with an air layer was irradiated with protons in simulation (B). We then placed 24 detectors on four sides of the phantom surface and estimated a 3D-DL dose. Third, a cylinder phantom with internal structures was used in simulation (C). The internal structures refer to hydroxyapatites (3.076 g/cm^3^) and glasses (1.8 g/cm^3^) of random size and were placed in random positions of the water phantom. The 36 × 12 detectors were placed around the phantom surface as shown in Figure 2 (Bottom). In this study, we positioned the detectors at a distance of 1 cm from the surface of the phantom. In the z-axis direction, the detectors were placed in rows without gaps. In the x-y plane, the detectors were placed at 10-degree intervals from the center. To avoid interference with the beam, detectors within 2 cm of the edge of the irradiated beam were removed. The proton beam irradiation was performed using the wobbler method, which involved spreading the beam perpendicularly to the direction of irradiation. In addition, we estimated the DL dose by utilizing the CT data as well as the readout currents. Note that, the CT data in this study refers to the density distribution of materials defined on Geant4. In each simulation, plastic scintillation detectors with the size of 10 mm × 10 mm × 3 mm were used to measure scattered particles. Plastic scintillators have a smaller reaction cross section for prompt gamma rays generated during treatment due to their low density. Additionally, their scalability and cost-effectiveness make them an appropriate choice for initial concept verification. In this paper, we defined the x-, y-, and z-axes as the proton beam direction, horizontal direction, and vertical direction, respectively.

**Table 1:** Summary of simulated conditions.

	**Phantom**	**Material**	**Proton shape**	**Detectors**	**Reconstruction**
Simulation (A)	Simple rectangular	Water	Pencil	24 × 2	2D
Simulation (B)	Rectangular w/ air layer	Water	Pencil	24 × 4	3D
Simulation (C)	Cylinder with internal structure	Water, hydroxyapatite, glass	Wobbler	36 × 12	3D

### 2.3. Deep learning models

**Figure 3:**
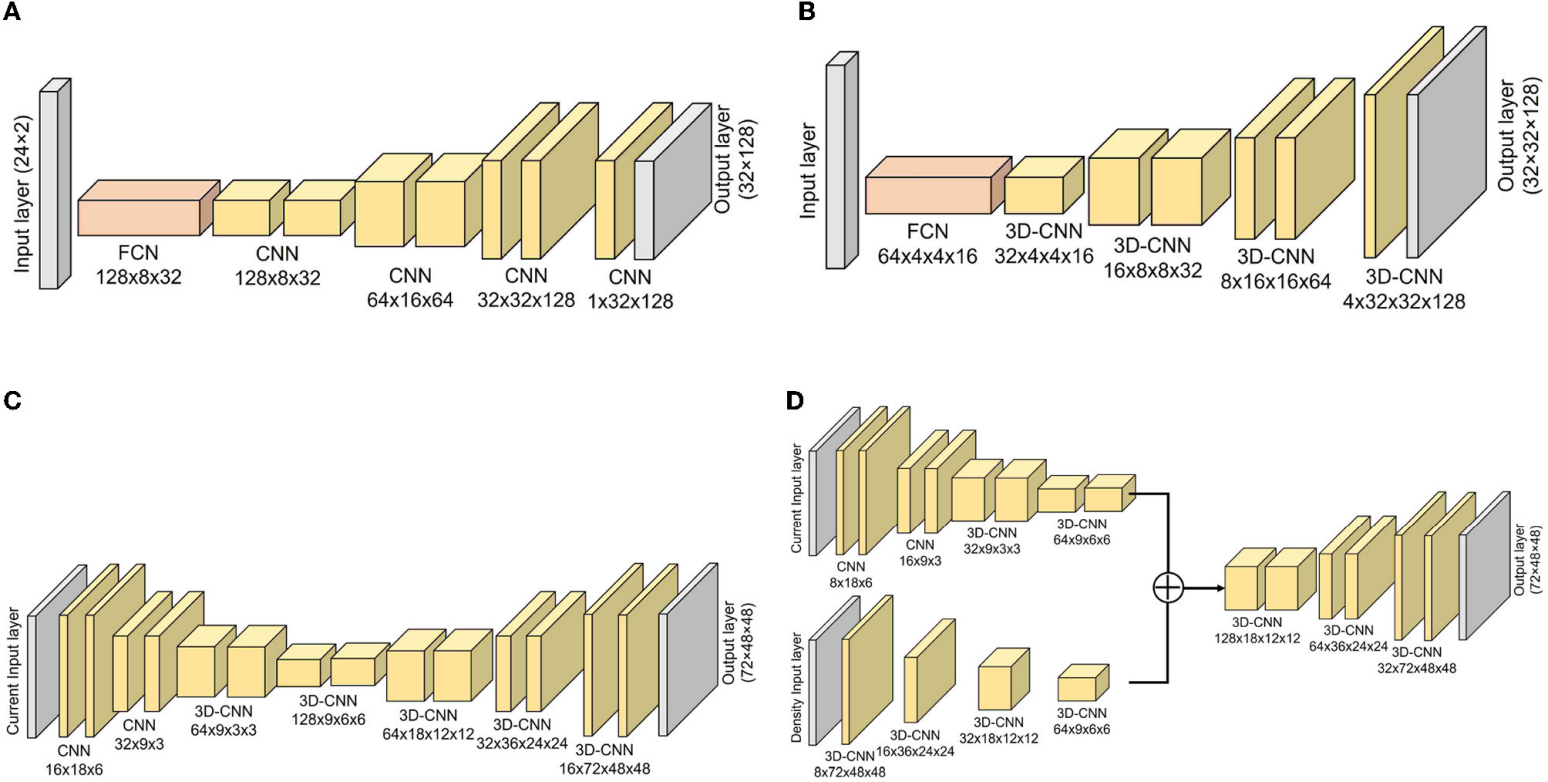
Implemented models for estimating DL dose. Models for simulation (A–C) are depicted. Additionally, the model used in simulation (D) with CT utilization are shown. **(A)** Simulation (A), **(B)** simulation (B), **(C)** simulation (C), and **(D)** simulation (C) with CT.

To estimate the DL dose, we used simple deep learning models which consist of fully connected networks (FCNs) and convolutional neural networks (CNNs). The models used in the respective simulations are shown in Figure 3. As the general generative model, the filter number was reduced along with upsampling, and the filter number in the first layer was adjusted to work well on our environment. A batch normalization layer and a leaky rectified linear unit (LeakyReLU) layer with α = 0.2 were applied after each CNN layer except for the last layer. After the last CNN, the hyperbolic tangent function (tanh) was used as an activation function. Given the current values from plastic scintillation detectors, our goal was to estimate the 2D- or 3D-DL dose. In simulation (C), we also utilized CT to estimate the DL dose. Mean squared error (MSE), which is one of the most common losses, was used as a loss function. Furthermore, the optimization of the model was performed using the Adam algorithm, with the hyperparameters of 200 epochs and a batch size of 4. These models were implemented with Pytorch (43), which is an open-source for machine-learning frameworks. In this study, the “GeForce RTX-2080Ti” graphics processing unit (GPU) processer was employed.

### 2.4. Database preparation

To estimate the DL dose by trained model, we prepared the datasets based on Geant4 simulation. The proton beam energy, beam center shift, and beam width were randomly determined for collecting various samples. The range of these parameters and the number of samples are summarized in Table 2. In simulation (B), the size and position of the air layer were randomly determined for each sample. Additionally, in simulation (C), hydroxyapatites and glasses of different sizes and shapes were randomly placed in the water phantom for each sample. Three to six internal structures were inserted into the water phantom with their sizes randomly determined between 5 cm and 20 cm. The shape of each internal structure was also randomly determined from an elliptical sphere, rectangle, or cylinder. In this study, the acquired dataset was partitioned into training, validation, and testing sets, comprising 64%, 16%, and 20% of the data amount, respectively. For simulation (A), data augmentation was performed by flipping the training and validation data twice along the y-axis. In the case of simulations (B) and (C), where the geometry complexity was higher, the training and validation data were augmented by flipping them along the y-axis and z-axis, respectively. In this study, we used the energy deposited into each detector as the current value, as it is a simplified model for a proof-of-principle test. Energy resolution and dark current are required to be considered for more detailed simulations.

**Table 2:** Details of database preparation.

	**Parameter ranges of proton beam**			**Sample number**		
	**Energy (MeV)**	**Beam center shift (y,z)**	**Beam width**	**Train**	**Validation**	**Test**
Simulation(A)	180–220	(Center± 4cm, No shift)	1cmϕ	1,747	437	273
Simulation(B)	180–220	(Center± 4cm, No shift)	1cmϕ	4,448	1,112	348
Simulation(C)	120–180	(Center± 16cm, Center± 16cm)	1–4cmϕ	3,888	976	305

Parameter ranges of proton beam on simulation and the number of samples are listed.

### 2.5. Quantitative evaluation indices

For evaluating the quality of the estimated DL dose, three evaluation indices were used: errors in the peak position for each direction (Δ*X*_peak_, Δ*Y*_peak_, Δ*Z*_peak_), root sum squared error Δ_dose_, and gamma passing rate $\Gamma_{{\vec{r}}_{e},{\vec{r}}_{r}}\left(\Delta D,\Delta d\right)$. First, errors in the peak position between the MC dose and DL dose were evaluated quantitatively since the position of the Bragg peak is important for verifying the effectiveness of proton therapy. Additionally to the peak position error, Δ_dose_ was evaluated. Δ_dose_ represents root sum squared error calculated in Eq. 2.


(2) $$\begin{array}{l} \Delta_{\text{dose}}=\sqrt{\Sigma_{i=1}^{n}{\left(I_{1}^{i}-I_{2}^{i}\right)}^{2}}, \end{array}$$

where *I*_1_ and *I*_2_ are MC dose and DL dose, respectively. *I*^*i*^ denotes the *i*^th^ pixel value. The Δ_dose_ is calculated by normalizing the absorbed dose of MC dose to 100 Gy. Finally, we computed 3D gamma index (γ), and evaluated the gamma passing rate. The 3D gamma index, which combines a dose difference and a distance-to-agreement, is calculated from the DL dose $D\left({\vec{r}}_{e}\right)$ and MC dose $D\left({\vec{r}}_{r}\right)$ as shown in Eq. 3 and Eq. 4.


(3) $$\begin{array}{l} \gamma \left({\vec{r}}_{r}\right)=\underset{{\vec{r}}_{e}}{\min}\left\{\Gamma_{{\vec{r}}_{e},{\vec{r}}_{r}}\left(\Delta D,\Delta d\right)\right\}, \end{array}$$

(4) $$\begin{array}{l} \Gamma_{{\vec{r}}_{e},{\vec{r}}_{r}}\left(\Delta D,\Delta d\right)=\sqrt{\frac{|D\left({\vec{r}}_{e}\right)-D\left({\vec{r}}_{r}\right){|}^{2}}{\Delta D^{2}}+\frac{|{\vec{r}}_{e}-{\vec{r}}_{r}{|}^{2}}{\Delta d^{2}}}, \end{array}$$

where Δ*D* and Δ*d* represent the acceptance criteria for the dose difference and distance-to-agreement, respectively. A voxel that satisfies $\gamma \left({\vec{r}}_{r}\right)<1$ is accepted, and the gamma passing rate represents the acceptance rate. In this study, the criteria for the gamma passing rate were 3 mm and 3%.

## 3. Results

### 3.1. Simulation (A): a simple rectangular phantom

**Figure 4:**
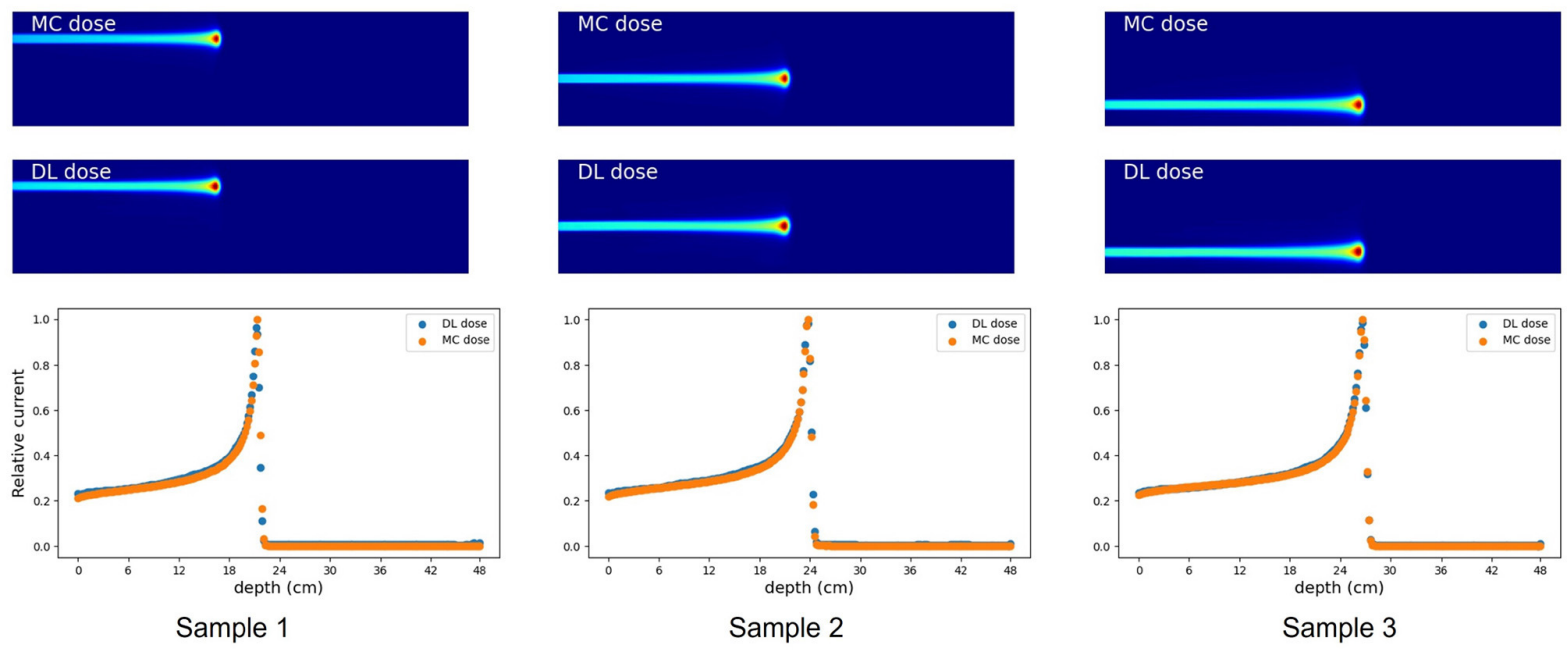
Verification result for three examples in simulation (A). MC dose **(Top)**, DL dose **(Middle)** and 1D projections of these dose ranges are depicted **(Bottom)**, respectively.

First, we evaluated the 2D-DL dose in simulation (A). Figure 4 shows three samples with different energies and irradiation positions. The 1D projection was created by integrating the pixel values in the sliced image. In all samples, the DL dose was estimated with high accuracy. Quantitatively, Table 3 shows the peak position errors between the MC dose and DL dose for simulation (A), (B), (C), and (C) with CT utilization. The reported values include the mean (Mean), standard deviation (Std), minimum (Min), and maximum (Max). As shown in Table 3 (top), we estimated the Bragg peak with an error of about 1.0 mm in the depth direction (X-axis) and 0.4 mm in the vertical direction (Y-axis). In addition, Table 4 show the evaluation result based on the Δ_dose_ and the gamma passing rate. Δ_dose_ equals 0.34 Gy for a total absorbed dose of 100 Gy, and the gamma passing rate was 99.7 %. In a simple situation, we successfully estimated the DL dose with the verification system.

**Table 3:** Errors in the peak position between the MC dose and DL dose.

**Simulation**	***ΔX***_*****peak*****_ **(mm)**				***ΔY***_*****peak*****_ **(mm)**				***ΔZ***_*****peak*****_ **(mm)**			
	**Mean**	**Std**	**Min**	**Max**	**Mean**	**Std**	**Min**	**Max**	**Mean**	**Std**	**Min**	**Max**
(A)	1.04	1.24	0.00	9.38	0.38	0.76	0.00	1.88	-	-	-	-
(B)	2.07	11.3	0.00	135	1.79	1.88	0.00	3.75	0.44	1.21	0.00	3.75
(C)	12.6	12.3	0.00	85.0	5.31	5.54	0.00	25.0	3.70	4.30	0.00	20.0
(C) with CT	9.30	12.6	0.00	95.0	5.54	5.48	0.00	35.0	3.52	3.97	0.00	20.0

**Table 4:** Summation of dose error (Δ_dose_) between the MC dose and DL dose, and Gamma passing rate based on gamma analysis Γ(3mm,3%) of these dose range.

**Simulation**	Δ_**dose**_				**Gamma passing rate**			
	**Mean (Gy)**	**Std (Gy)**	**Min (Gy)**	**Max (Gy)**	**Mean (%)**	**Std (%)**	**Min (%)**	**Max (%)**
(A)	0.34	0.16	0.11	1.37	99.7	0.60	92.4	99.9
(B)	0.31	0.18	0.11	1.64	98.0	3.59	72.4	100
(C)	2.66	1.60	0.69	9.46	32.5	15.9	0.00	82.8
(C) with CT	2.26	1.23	0.65	8.67	39.5	19.1	0.65	83.7

### 3.2. Simulation (B): a rectangular water phantom with an air layer

**Figure 5:**
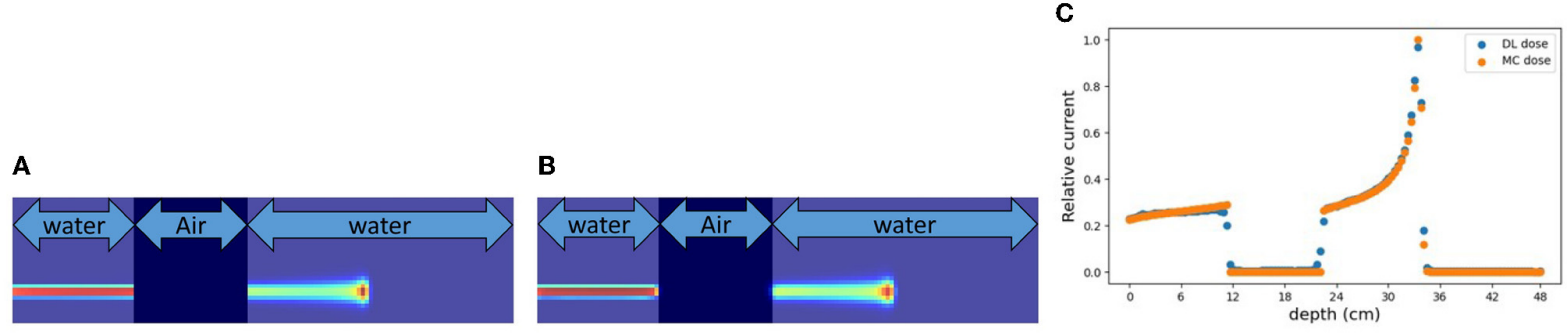
An example of verification result in simulation (B). **(A)** MC dose (slice), **(B)** DL dose (slice), and **(C)** 1D projection of these doses.

As part of simulation (B), a water phantom with an air layer was used and the 3D-DL dose was estimated. Figure 5 shows an example of the estimating result. Most samples accurately estimated the location of the Bragg peak and the air layer such as Figure 5. Meanwhile, the width of the air layer gets slightly mis-predicted in some cases due to the statistical noise of output current. As shown in Table 3 (second row), we estimated the Bragg peak with an error of about 2.1 mm in the depth direction. Δ_dose_ and the gamma passing rate were 0.31 Gy and 98.0 %, respectively. These evaluation results indicate that the estimation quality was sufficient for elementary situations such as simulation (B). Compared to simulation (A), with the increase in phantom complexity, it became more difficult to estimate the position of the Bragg peak. In simulation (B), the standard deviation of Δ*X*_peak_ was larger than simulation (A). This was attributed to the presence of an air layer intersecting the Bragg peak, leading to a notable increase in error resembling an outlier. Notably, Table 3 presents the average error to be a few mm, whereas the maximum error reached up to 135 mm.

### 3.3. Simulation (C): a cylindrically shaped water phantom comprising of internal structures

**Figure 6:**
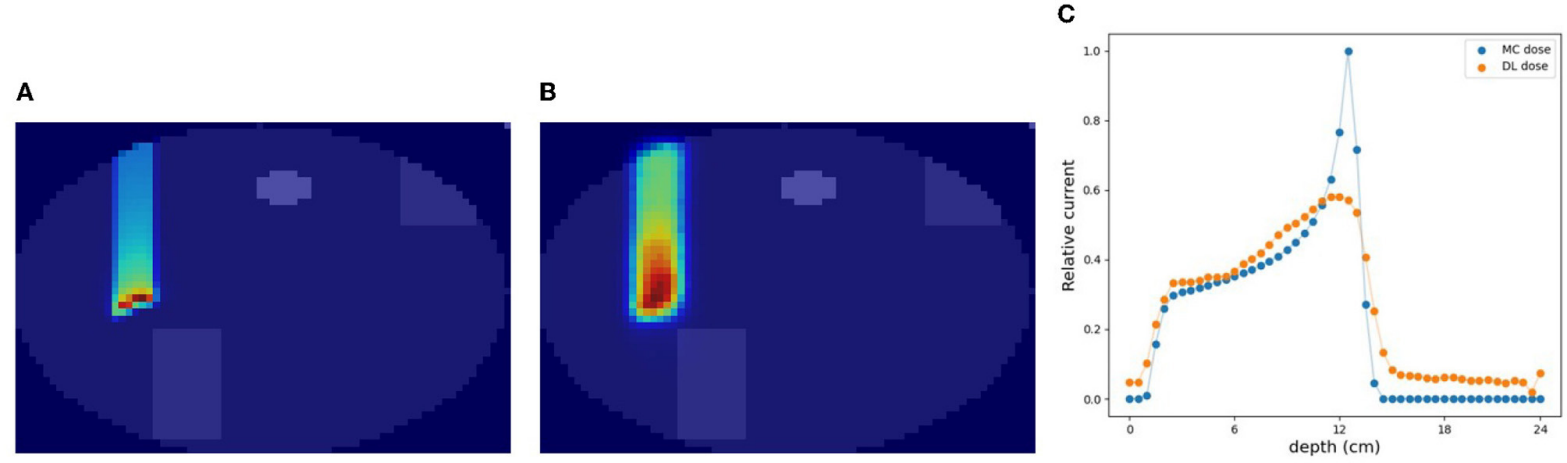
An example of verification result in simulation (C). **(A)** MC dose (slice), **(B)** DL dose (slice), and **(C)** 1D projection of these doses.

**Figure 7:**
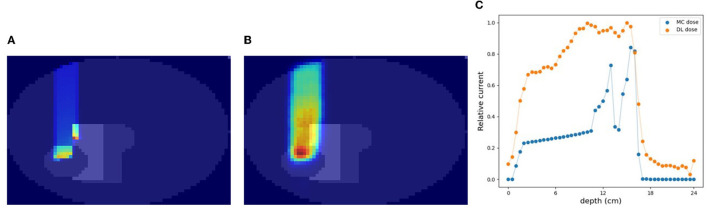
Another example of verification result in simulation (C). **(A)** MC dose (slice), **(B)** DL dose (slice), and **(C)** 1D projection of these doses.

A cylinder phantom with an internal structure was used and the 3D-DL dose was estimated in simulation (C). Two verification examples are shown in Figures 6, 7. The MC dose and DL dose were overlaid on the corresponding CT. Figure 6 shows an example where the internal structure did not exist between the incident and the target area. Thus, the 1D projection of MC dose had a shape similar to the irradiation of a water phantom without an internal structure. On the other hand, Figure 7 shows an example where the proton beam directly irradiates the internal structures. The proton beam range was shortened as a consequence of reacting with a material that was denser than water. In the MC dose, the dose exhibited sharp changes at the edges of the internal structure. In the current simulation, we considered multiple scattering effects, but the sharp appearance was mainly due to the relatively coarse resolution of 5.0 mm/pixel. As a result of the estimation, the DL dose was roughly estimated in both samples. However, the density information was crucial to estimate the details of the DL dose as described in Section 3.5. Quantitatively, we estimated the Bragg peak with an error of about 12.6 mm in the depth direction as described in Table 3 (third row). In general, even for rough monitoring, the dose range error is required to be improved by better than 10 mm. In the following section, we utilized the CT data as well as the output current to reduce the error.

### 3.4. Simulation (C) with CT data

**Figure 8:**
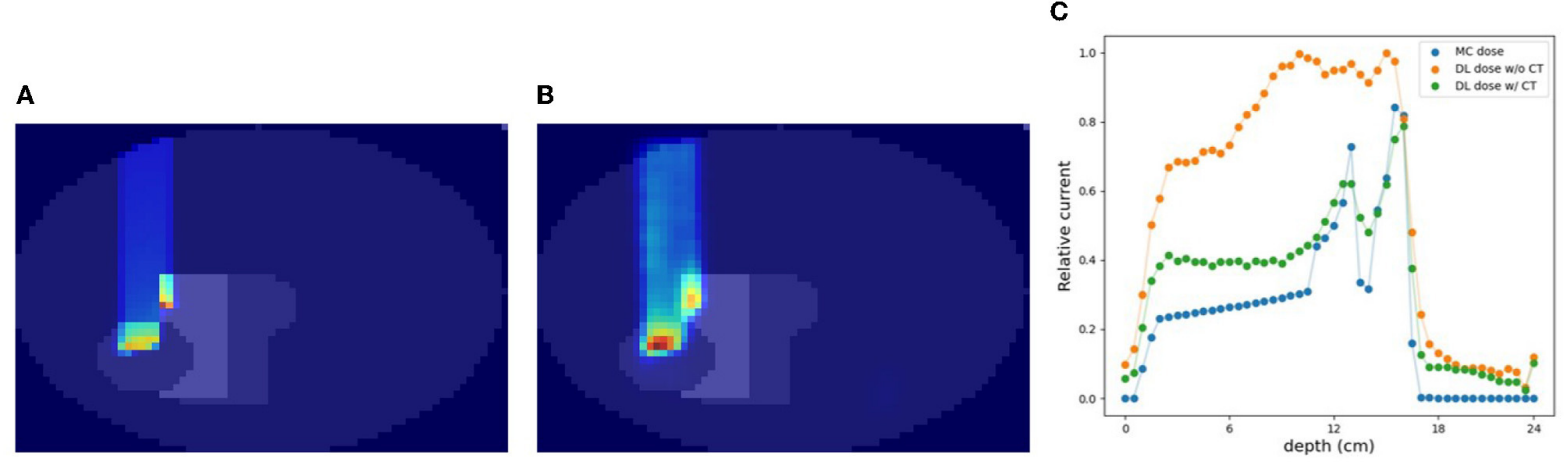
An example of verification result in simulation (C) with CT. **(A)** MC dose (slice), **(B)** DL dose (slice), and **(C)** 1D projection of these doses.

In this section, we estimated the DL dose with the density distribution as well as the current values from detectors in simulation (C). Figure 8 shows the results of utilizing CT data for the same sample as in Figure 7. Compared with Figure 7, the estimation quality of DL dose was improved. Quantitatively, we estimated the Bragg peak with an error of about 9.3 mm in the depth direction as shown in Table 3 (bottom row). Additionally, the Δ_*dose*_ values and the peak position error in depth were improved by utilizing CT data.

### 3.5. Effectiveness of CT utilization

**Figure 9:**
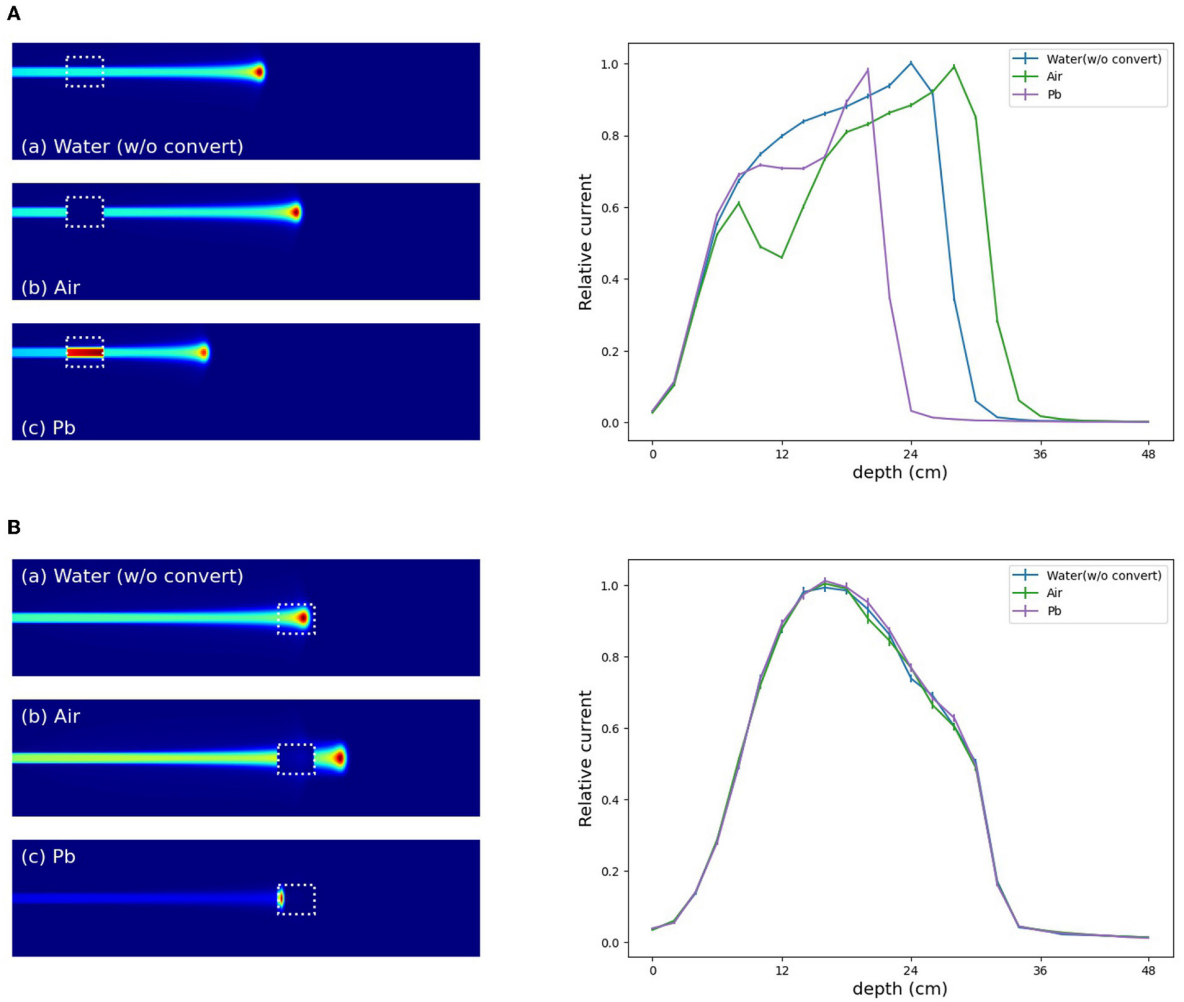
Change in current value with substitution of phantom material. The MC dose in (a) without replacing, (b) replacing to air, and (c) replacing to Pb. Output currents are summarized (right). **(A)** Substitution of phantom material in front of the Bragg peak. **(B)** Substitution of phantom material around the Bragg peak.

**Figure 10:**
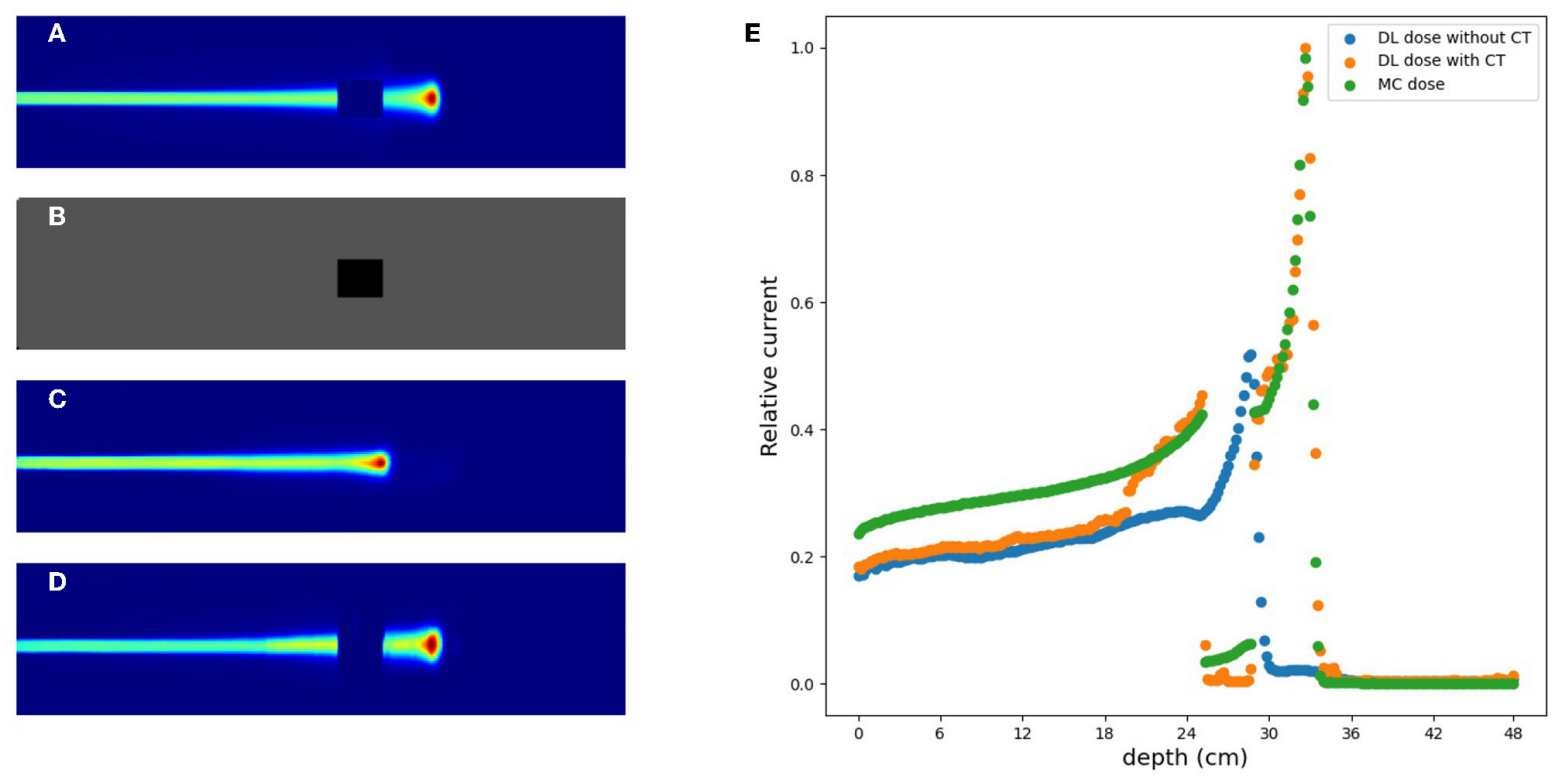
An example of verification results with and without CT utilization. **(A)** MC dose (3D), **(B)** CT image, **(C)** DL dose without CT, **(D)** DL dose with CT, and **(E)** 1D projection of MC dose and DL dose.

In the verification system, we measured the scattered protons from a phantom. The distribution of scattered protons roughly reflects the MC dose. However, measured values do not reflect the MC dose around the Bragg peak since the protons we observe are mainly scattered in the front part of the phantom. For confirming the effect, we substituted a part of the phantom for another material (air and Pb) in experiment (A). The area surrounded by the square was replaced with other materials and the measured current values were compared in Figure 9. The shape of the observed currents differed significantly in the case where the material of the front part of the phantom was substituted as shown in Figure 9 (top). However, the current values were consistent within the error range as shown in Figure 9 (bottom right), while the shape and position of the Bragg peak vary with the substitution of materials. Since the protons near the Bragg peak has only small energy and thus can be easily absorbed, these protons do not affect the current values. Therefore, to verify the precise dose range, especially around the Bragg peak, density information such as CT is necessary to be used as a prior. For verifying the effectiveness of CT utilization, a dataset was prepared with a part of the water phantom replaced by air or Pb and was used for training the deep learning model. Figure 10 shows the estimated result with and without CT utilization. Without CT, the DL dose for the air part has a significant error. Meanwhile, the use of CT improved the estimation results.

### 3.6. System optimization

**Figure 11:**
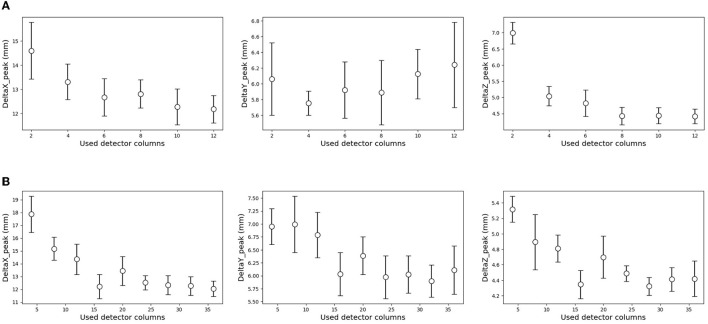
Effect of the reduced number of detectors on the estimation results. The result of a decrease in the horizontal direction **(A)**, and vertical direction **(B)**. The error range was defined by a standard deviation of 10 times estimation result in each condition. **(A)** Reducing the number of detectors in the horizontal direction. **(B)** Reducing the number of detectors in the vertical detector.

As shown in Figure 2 (bottom), we temporarily placed 36 × 12 detectors surrounding the phantom in simulation (C). To optimize the system, a similar simulation was performed in this section with a reduced number of detectors. The details of the used detector ID are described in the Supplementary material. For simplicity, the current encoded part of the model in Figure 3 (bottom right) was replaced by FCN. Figure 11A shows the result for reducing the number of detectors in the horizontal direction (x-y plane). Peak position errors in all directions deteriorated while reducing the number of detectors in the horizontal direction. Also, the Δ*X*_peak_ and Δ*Y*_peak_ were worsened as the number of detectors in the vertical direction (z-axis) was reduced as shown in Figure 11B. The vertical peak position is considered to be calculated from the ratio of the current values in each row. Therefore, the impact of reducing the number of rows may be small when the number of detectors per row is sufficient. To summarize the results, reducing the number of detectors from 36 to 18 in the horizontal direction and from 12 to 6 in the vertical direction was not considered to have a significant impact.

## 4. Discussion

### 4.1. Estimation quality limitation

In this chapter, we discuss the limitation of estimation quality in simulation (C). In simulation (C), which differed from simulations (A) and (B), protons were irradiated from the short-axis side of the ellipsoidal phantom. This arrangement resulted in a long distance between the irradiated area and the detector, making it challenging to detect scattered protons. Furthermore, there are a few internal structures in the phantom. Thus, simulation (C) exhibited inferior performance compared to simulations (A) and (B). As demonstrated in Section 3.5, the measured currents did not reflect the dose range around the Bragg peak since the protons we observe are mainly scattered in the front part of the phantom. Without CT utilization, it is difficult to estimate the exact Bragg peak position. Therefore, the model was trained to produce a blurred image so that the average dose range was obtained. As a result, the DL dose exhibited blurriness even in cases such as Figure 6, where the proton beam did not traverse the internal structure.

The estimation performance of our system was constrained by factors including the deep learning model, the training dataset, and the characteristics of the imaging system. In the current simulation, the estimation performance was partially compensated for the unavailability of dose information near the Bragg peak, owing to the accurate geometry acquisition. Consequently, the estimation performance in this paper was primarily limited by the training data and model. To mitigate computational costs, 1.0 × 10^7^ protons, equivalent to a 0.1-second irradiation of a clinical beam, were irradiated in this study, resulting in statistical errors. Moreover, the simplicity and compactness of the model further restrict the accuracy of estimating the 3D-DL dose.

In this study, we implemented deep learning models that combined CNN and FCN. Nonetheless, under challenging conditions such as simulation (C), a simplistic CNN model exhibited limited estimation performance. The present model performed convolutions on the current values in two dimensions, disregarding the three-dimensional detector position. As a future prospect, we are considering the incorporation of a module to estimate the Bragg peak position based on the three-dimensional detector position using a centroid method. Furthermore, we anticipate that further improvements in estimation performance can be achieved by augmenting the dataset size and constructing a large-scale model utilizing residual networks (ResNet) (44).

### 4.2. Comparison with other methods

In this section, we compared the proposed verification system with conventional methods: annihilation gamma rays measurement by an in-beam PET (16), prompt gamma rays measurement by a Compton camera (25), luminescence imaging by a charge-coupled device (CCD) (31), and secondary-electron-bremsstrahlung (SEB) imaging by a pinhole gamma camera (45). First, according to the in-beam PET, the image was reconstructed within 6 seconds of beam irradiation, and the range agreement was within 1 mm. While possessing sufficient estimation quality, the PET system tends to be expensive and large. Regarding a Compton camera, prompt gamma rays have been measured by a set of scintillation detectors. The average peak position error was 6.2 mm for a simple poly methyl methacrylate (PMMA) phantom. While a Compton camera has the potential to verify the dose range in real-time due to the prompt gamma rays measurement, the reconstructed image had significant artifacts. Concerning the luminescence image by a cooled CCD camera, the image was reconstructed within 1 second by U-net, and the range agreement was about 0.35 mm. Additionally to the high speed and accurate reconstruction, a CCD camera has a simple configuration and cost-effectiveness. However, it is difficult to verify the proton dose range in the human body as the luminescence is faint. Finally, SEB imaging based on a pinhole gamma camera also has the potential of luminescence imaging. The spatial resolution (FWHM) 11.0 cm apart from the camera was 3.23 mm in simple geometry. Although the SEBs easily leak outside the body due to their relatively higher energy than visible light, there is a challenge with statistics owing to the collimation of the pinhole gamma camera. On the other hand, the proposed verification system measures the scattered protons by scintillation detectors. The image was reconstructed in 3.1 ms, and the average peak position error was 1.0 mm for the simple situation. However, the dose range error deteriorated to 9.3 mm for the complex phantom. To summarize then, the proposed verification system is suitable for confirming the proton irradiation region in real-time with a simple and small setup. In addition, flash therapy (46–48), in which protons are irradiated with very high intensity for a short period, has attracted attention in recent years. Since high-rate tolerance is required to observe the dose range in flash therapy, the proposed verification system based on the current readout will be more in demand hereafter due to its high flux rate tolerance.

Fast dose calculation (49, 50) refers to a technique that utilizes CT to expedite MC simulations for accurate treatment planning. While it enables efficient calculation of the treatment effect, it remains a theoretical estimation. Therefore, it is crucial to incorporate monitoring methods such as our proposed approach or PET, to validate the alignment between the administered beam and the intended treatment.

### 4.3. Challenges for practical use

There are some challenges to our system for practical use. First, deviation in particle process between actual measurement and simulation may deteriorate the estimation quality. This matter is resolved by increasing the accuracy of simulation and/or fine-tuning using actual measured data. In the case of fine-tuning, a model that has already been trained based on simulation data is additionally trained with a small amount of measured data. Second, it is necessary to consider that each patient has a different body size and shape. Although the phantom size and shape are fixed in the simulations of this paper, we should prepare a variety of phantoms on Geant4 for practical use. Third, a system designed to cover the entire human body may result in beam inhibition. To address this issue, we propose utilizing a stretchable band and fixing the detectors by attaching them to the band. As discussed in Sec. 3.6, the estimation performance could be maintained even with a quarter of the number of detectors. Thus, we expect the detectors to be placed sparsely so that monitoring will not interfere with the beam. In addition, the scattered protons we measure lose most of their energy while leaking out of the body. Since the statistically low percentage of scattered protons, we have chosen to place the detector as close to the body surface as possible. However, we acknowledge that the detector setup may be challenging, and we will explore the possibility of placing the detector at a distance from the body surface through simulations. Finally, while the geometry is measured prior to treatment, the distribution within the body undergoes changes during actual irradiation due to factors like breathing-induced body movements. Consequently, the imaging system imposes limitations on the actual measurement. To enhance estimation accuracy, the strategy described in Section 4.1 will be implemented.

## 5. Conclusion

We conducted three stepwise simulations to demonstrate the feasibility of the as-proposed verification system by estimating the DL dose. As shown in simulations (A) and (B), we successfully estimated the DL dose under the simple situation within a 2 mm peak position error. In simulation (C), the position error of the Bragg peak was 12.6 mm for a cylinder phantom with internal structures. By utilizing CT, the peak position error was improved to 9.3 mm. These simulation results suggest the feasibility of the proposed verification system. In the future, we plan to conduct the experimental verification for human body phantom to optimize the geometry as well Additionally, to contribute a flash therapy, we will quantitatively evaluate the flux rate tolerance.

## Data availability statement

The raw data supporting the conclusions of this article will be made available by the authors, without undue reservation.

## Author contributions

SS collected the data, implemented deep learning models, and wrote the first manuscript. JK supervised the project. All authors contributed to the article and approved the submitted version. All authors contributed to the conception and design of the study.
