# Supplementary material for: A simulation study of in-beam visualization system for proton therapy by monitoring scattered protons
Source: Front Med (Lausanne). 2023 Jul 14;10:1038348. doi: 10.3389/fmed.2023.1038348 (PMC10375415; doi:10.3389/fmed.2023.1038348)
Supplement: Supplementary file 1 [file Data_Sheet_1.PDF]

Supplementary Material

1 GAMMA PASSING RATE

In this study, we evaluated the estimation dose distribution by gamma passing rate Γ(3 mm,3%). Since the grid resolution was $5 \times 5 \times 5$ mm^3 in simulation (C), it may have been an unfavorable evaluation. Therefore, the acceptance criteria were changed to 5 mm, 7 mm, and 10 mm for re-evaluation, as shown in Table. S1.

Table S1. Gamma passing rate of dose distribution. Gamma analysis results Γ(3mm,3%) for simulation (A), (B), (C), and (C) with CT utilization are listed. The reported values include the mean (Mean), standard deviation (Std), minimum (Min) and maximum (Max).

simulation	Γ(5mm,3%) (%)				Γ(7mm,3%) (%)				Γ(10mm,3%) (%)			
	Mean	Std	Min	Max	Mean	Std	Min	Max	Mean	Std	Min	Max
(A)	99.8	0.33	94.6	99.9	99.8	0.26	95.9	99.9	99.7	0.29	97.5	99.9
(B)	98.8	2.88	75.0	100.0	99.0	2.58	77.2	100.0	99.1	2.35	80.2	100.0
(C)	45.8	19.8	0.81	89.4	55.7	21.4	1.63	94.9	65.8	22.1	4.07	98.1
(C) with CT	54.7	21.7	1.85	93.6	66.1	22.1	3.42	97.4	76.9	20.7	6.01	99.3

Even though the acceptance criteria were changed from 3 mm to 10 mm, the value of the gamma passing rate was about 77%. Therefore, further improvement in accuracy is necessary for precise monitoring.

2 PROCEDURES FOR REDUCING DETECTORS IN SYSTEM OPTIMIZATION

To optimize the proposed verification system, we reduced the number of scintillation detectors in simulation (C) with CT. The estimation quality depends on the columns and rows of the detectors used. Therefore, the column IDs used for horizontal detector reduction are shown in Table. S2, and row IDs for vertical detector reduction are shown in Table. S3. The column IDs and row IDs for horizontal and vertical directions are depicted in Fig. 2.

Table S2. Detector IDs used for horizontal detector reduction.

used columns	0	1	2	3	4	5	6	7	8	9	10	11	12	13	14	15	16	17
all columns	○	○	○	○	○	○	○	○	○	○	○	○	○	○	○	○	○	○
32 columns	○	○	○	○		○	○	○	○	○	○	○	○	○		○	○	○
28 columns	○	○		○	○	○	○		○	○	○		○	○	○	○		○
24 columns	○		○	○		○	○		○	○		○	○		○	○		○
20 columns	○		○		○		○	○		○		○	○		○		○	
16 columns	○		○			○		○			○			○		○		○
12 columns	○			○			○			○			○			○		
8 columns	○				○					○					○			
4 columns					○										○			

used columns	18	19	20	21	22	23	24	25	26	27	28	29	30	31	32	33	34	35
all columns	○	○	○	○	○	○	○	○	○	○	○	○	○	○	○	○	○	○
32 columns	○	○	○	○		○	○	○	○	○	○	○	○	○		○	○	○
28 columns	○	○		○	○	○	○		○	○	○		○	○	○	○		○
24 columns	○	○		○	○		○	○		○	○		○	○		○	○	
20 columns	○		○		○		○	○		○		○	○		○		○	
16 columns		○		○		○			○		○		○		○	○		
12 columns	○			○			○			○			○			○		
8 columns	○				○					○					○			
4 columns					○										○			

Table S3. Detector IDs used for vertical detector reduction.

used rows	0	1	2	3	4	5	6	7	8	9	10	11
all rows	○	○	○	○	○	○	○	○	○	○	○	○
10 rows	○	○	○		○	○	○	○		○	○	○
8 rows	○		○	○		○	○		○	○		○
6 rows		○		○		○	○		○		○	
4 rows			○		○			○		○		
2 rows				○					○			

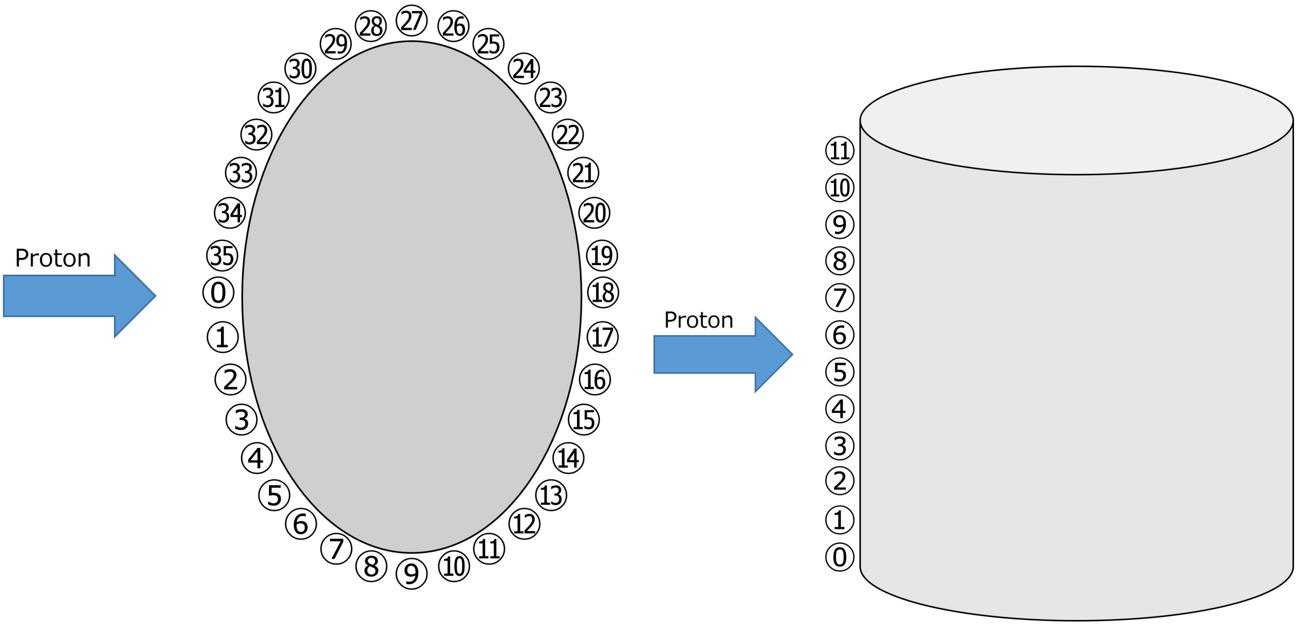

Figure 2a. Columns ID (horizontal) **Figure 2b.** Rows ID (vertical)

Figure 2. Detector IDs for horizontal and vertical direction.

3 EFFECT OF PATIENT MORPHOLOGY

In this study, we utilized density distribution such as the CT for treatment planning. However, the density distribution during treatment may be different from the treatment planning. To assess the impact of this difference, we evaluated the effect of changing the internal structure on the performance of our deep learning model. Specifically, we randomly rotated the internal structure within ± 5 degrees and translated it within ± 1cm, and input the modified CT into the model. The quantitative evaluation of the inference results is presented in Tables S4 and S5. Consequently, the inference performance deteriorated slightly, since the phantoms used in this study consisted of a simple combination of spheres and rectangles. Thus, a more comprehensive evaluation such as a human phantom should be conducted in future studies.

Table S4. Errors in the peak position between the estimated dose range and ground truth.

simulation	ΔX_{peak}(mm)				ΔY_{peak}(mm)				ΔZ_{peak}(mm)			
	Mean	Std	Min	Max	Mean	Std	Min	Max	Mean	Std	Min	Max
(A)	1.04	1.24	0.00	9.38	0.38	0.76	0.00	1.88	-	-	-	-
(B)	2.07	11.3	0.00	135	1.79	1.88	0.00	3.75	0.44	1.21	0.00	3.75
(C)	12.6	12.3	0.00	85.0	5.31	5.54	0.00	25.0	3.70	4.30	0.00	20.0
(C) with CT	9.30	12.6	0.00	95.0	5.54	5.48	0.00	35.0	3.52	3.97	0.00	20.0
(C) with deformation CT	9.84	12.7	0.00	90.0	5.11	5.34	0.00	30.0	3.70	4.04	0.00	20.0

Table S5. Summation of dose error (Δ_{dose}) between the estimated dose range and ground truth and Gamma passing rate based on gamma analysis Γ(3mm,3%) of dose distribution.

simulation	Δ_{dose}				Gamma passing rate			
	Mean (Gy)	Std (Gy)	Min (Gy)	Max (Gy)	Mean (%)	Std (%)	Min (%)	Max (%)
(A)	0.34	0.16	0.11	1.37	99.7	0.60	92.4	99.9
(B)	0.31	0.18	0.11	1.64	98.0	3.59	72.4	100
(C)	2.66	1.60	0.69	9.46	32.5	15.9	0.00	82.8
(C) with CT	2.26	1.23	0.65	8.67	39.5	19.1	0.65	83.7
(C) with deformation CT	2.32	1.25	0.63	8.16	39.3	18.5	0.74	87.4
